# Modulation of CD4^+^ T Cell-Dependent Specific Cytotoxic CD8^+^ T Cells Differentiation and Proliferation by the Timing of Increase in the Pathogen Load

**DOI:** 10.1371/journal.pone.0000393

**Published:** 2007-04-25

**Authors:** Fanny Tzelepis, Pedro M. Persechini, Mauricio M. Rodrigues

**Affiliations:** 1 Centro Interdisciplinar de Terapia Gênica (CINTERGEN), Universidade Federal de São Paulo-Escola Paulista de Medicina, São Paulo, Brazil; 2 Departamento de Microbiologia, Imunologia e Parasitologia, Universidade Federal de São Paulo-Escola Paulista de Medicina, São Paulo, Brazil; 3 Instituto de Biofísica Carlos Chagas Filho, Centro de Ciências da Saúde, Universidade Federal do Rio de Janeiro, Ilha do Fundão, Rio de Janeiro, Brazil; University of California, San Francisco, United States of America

## Abstract

**Background:**

Following infection with viruses, bacteria or protozoan parasites, naïve antigen-specific CD8^+^ T cells undergo a process of differentiation and proliferation to generate effector cells. Recent evidences suggest that the timing of generation of specific effector CD8^+^ T cells varies widely according to different pathogens. We hypothesized that the timing of increase in the pathogen load could be a critical parameter governing this process.

**Methodology/Principal Findings:**

Using increasing doses of the protozoan parasite *Trypanosoma cruzi* to infect C57BL/6 mice, we observed a significant acceleration in the timing of parasitemia without an increase in mouse susceptibility. In contrast, in CD8 deficient mice, we observed an inverse relationship between the parasite inoculum and the timing of death. These results suggest that in normal mice CD8^+^ T cells became protective earlier, following the accelerated development of parasitemia. The evaluation of specific cytotoxic responses *in vivo* to three distinct epitopes revealed that increasing the parasite inoculum hastened the expansion of specific CD8^+^ cytotoxic T cells following infection. The differentiation and expansion of *T. cruzi*-specific CD8^+^ cytotoxic T cells is in fact dependent on parasite multiplication, as radiation-attenuated parasites were unable to activate these cells. We also observed that, in contrast to most pathogens, the activation process of *T. cruzi*-specific CD8^+^ cytotoxic T cells was dependent on MHC class II restricted CD4^+^ T cells.

**Conclusions/Significance:**

Our results are compatible with our initial hypothesis that the timing of increase in the pathogen load can be a critical parameter governing the kinetics of CD4^+^ T cell-dependent expansion of pathogen-specific CD8^+^ cytotoxic T cells.

## Introduction

MHC class Ia-restricted CD8^+^ T cells are important mediators of the adaptive immune response against infections caused by intracellular microorganisms. Following infection with certain viruses, bacterias or parasites, naïve antigen-specific CD8^+^ T cells go through a process of fast differentiation and proliferation, generating effector cytotoxic cells (expansion phase). These effector cells circulate between lymphoid and non-lymphoid tissues to restrain the multiplication of the infectious pathogen. Following pathogen elimination, the number of specific effector CD8^+^ T cells is drastically reduced (contraction phase) and the establishment of a long-lived population of memory T cells responsible for perpetuating immunity against re-infection takes place. This kinetics of effector CD8^+^ T cell expansion, contraction and establishment of a memory population has been fairly well reproduced and it is being thoroughly studied in a number of experimental models, including virus, bacterias and protozoan parasites (reviewed in ref. 1–3).

Using these experimental models, it was possible to establish that specific CD8^+^ T cells differentiate and proliferate very quickly reaching a peak between days 4 and 8 after immunization with either lymphocytic choriomeningitis virus (LCMV), influenza virus, vaccinia virus, *Listeria monocytogenes* or *Plasmodium yoelli* [4]–[9]. Recent studies in mice infected with *Toxoplasma gondii*, *Mycobacterium bovis* bacille Calmette-Guerin (BCG), *Trypanosoma cruzi* and *Salmonella typhimurium* described significantly different kinetics of differentiation and proliferation of specific CD8^+^ T cells. In the case of *T. gondii*, CD8^+^ T cells specific for a transgenic epitope became detectable only 10 days after challenge, and the maximum number of epitope-specific T cells peaked at day 23 [10]. Similarly, in mice injected with recombinant *S. typhimurium* or BCG the peak response to the transgenic epitope was day 21^st^ or 30^th^ following challenge, respectively [11], [12].

We recently described the kinetics of parasite-specific cytotoxic CD8^+^ T cell responses following mouse infection with the human protozoan parasite *Trypanosoma cruzi* [13]. An interesting finding was that the initial inoculum of *T. cruzi* did not drive the differentiation and proliferation of effector CD8^+^ T cells. The expansion phase of specific splenic CD8^+^ T cells occurred after *in vivo* multiplication of parasites, between days 9 and 15 after i.p. challenge of C57BL/6 mice with 10^4^ parasites of the Y strain. More recently, Martin *et al*., (2006) confirmed and extended our results in studies using different *T. cruzi* strains, which revealed that the peak of parasite epitope-specific CD8^+^ T cells could vary from 14 to 24 days post-infection [14].

The results obtained during *T. gondii*, BCG, *S. typhimurium* or *T. cruzi* infections sharply differed from the observations made in mice infected with LCMV, vaccinia, influenza, *L. monocytogenes* or *P. yoelli* and raised questions on the possible mechanisms controlling the kinetics of differentiation and proliferation following infection with different pathogens.

One mechanism that could influence the kinetics of specific CD8^+^ T cells differentiation and proliferation is the amount of antigen and parasite-derived adjuvant both of which accumulate during the infection. Immediately after the initial infectious inoculum, the amount of antigen and parasite adjuvant available for CD8^+^ T-cell priming may be limited, however both will increase after pathogen multiplication, and then may reach a certain threshold necessary to promote the maturation of antigen presenting cells (APC) - in the case of the adjuvant molecule - and trigger the activation of naïve CD8^+^ T cells, in the case of the antigen. If this hypothesis is correct, the timing of increase of pathogen adjuvant/antigen would be a key parameter governing the process of CD8^+^ T cell differentiation and expansion.

The aim of the present study was to determine whether modulation of the parasite load within a certain period of time can in fact alter the activation of effector/protective CD8^+^ T cells. For this purpose, we used an experimental model where C57BL/6 mice were challenged with different doses of parasite of the Y strain of *Trypanosoma cruzi*. This strategy allowed us to modulate the timing of increase in the parasite load and determine the effect it may have on the *in vivo* differentiation and proliferation of effector/protective CD8^+^ T cells. Using this experimental model, we were able to lend support to the hypothesis that the timing of accumulation of the parasite load can be a key factor influencing the differentiation and proliferation of CD4^+^ T cell-dependent specific CD8^+^ cytotoxic T cells following infection with a human pathogen.

## Methods

### Mice and parasites

Female 8 to 10-week-old wild type (WT) C57BL/6, CD8α deficient C57BL/6 (CD8 KO), MHC-II deficient C57BL/6 (MHC-II KO), CD4 deficient C57BL/6 (CD4 KO), p40 deficient C57BL/6 (IL-12 KO), wild type 129 and 129 deficient for the IFN-I receptor (IFN-I receptor KO) were obtained from University of São Paulo. Perforin deficient C57BL/6 (Perforin KO) mice were bred on our own facility.

Parasites of the Y strain of *T. cruzi* were used in this study [13]. Bloodstream trypomastigotes were obtained from the plasma of A/Sn mice infected 7 days earlier. The concentration of parasites was adjusted and each mouse was inoculated intraperitoneally (i.p.) with 0.2 mL containing the indicated amount of trypomastigotes. Parasite development was monitored by counting the number of bloodstream trypomastigotes in 5 µl of fresh blood collected from the tail vein [13]. When the parasitemia was above 10^5^ trypomastigotes per mL, blood samples were diluted and the number of parasites estimated with the aid of a hemacytometer. Radiation-attenuated parasites were obtained by exposing them to gamma-irradiation (100 krads).

### Immunological assays

For the *in vivo* cytotoxicity assays, C57BL/6 splenocytes were divided into two populations and labeled with the fluorogenic dye carboxyfluorescein diacetate succinimidyl diester (CFSE Molecular Probes, Eugene, Oregon, USA) at a final concentration of 5 µM (CFSE_high_) or 0.5 µM (CFSE_low_). CFSE_high_ cells were pulsed for 40 min at 37°C with 1 µM of the H-2K^b^ ASP-2 peptide (VNHRFTLV), or TsKb-18 (ANYKFTLV) or TsKb-20 (ANYDFTLV). CFSE_low_ cells remained unpulsed. Subsequently, CFSE_high_ cells were washed and mixed with equal numbers of CFSE_low_ cells before injecting intravenously (i.v.) 15 to 20×10^6^ total cells per mouse. Recipient animals were mice that had been infected or not with *T. cruzi*. Spleen cells of recipient mice were collected 20 h after transfer, fixed with 3.7% paraformaldehyde and analyzed by fluorescence-activated cell sorting (FACS), using a Facscalibur Cytometer (BD, Mountain View, CA). The percentage of specific lysis was determined using the formula:
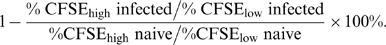
1


The ELISPOT assay for enumeration of Interferon-gamma (IFN-γ) producing cells was performed essentially as described earlier [15].

### Statistical analysis

The values of were compared by One-Way Anova followed by Tukey HSD tests available at the site http://faculty.vassar.edu/lowry/VassarStats.html. The LogRank test was used to compare the mouse survival rate after challenge with *T. cruzi*. The differences were considered significant when the *P* value was <0.05.

## Results

**Figure 1:**
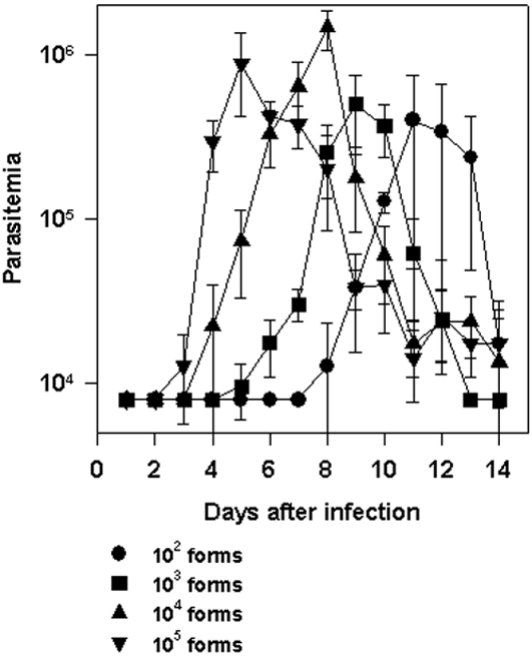
Trypomastigote-induced parasitemia in C57BL/6 mice challenged with different doses of trypomastigotes of *T. cruzi.* C57BL/6 mice were infected i.p. with 10^2^,10^3^, 10^4^ or 10^5^ bloodstream trypomastigotes of the Y strain of *T. cruzi*. Parasitemia was followed daily from days 0 to 14 after challenge. The results represent the mean of 5–6 mice±SD. At the peak of infection, the parasitemia of mice infected with each different dose was compared by One-way Anova and Tukey HSD tests. The results of the comparisons were as follows: i) 10^2^×10^3^, Non-Significant (NS); ii) 10^2^×10^4^, *P*<0.01; iii) 10^2^×10^5^, *P*<0.01; iv) 10^3^×10^4^, *P*<0.05; v) 10^3^×10^5^, NS; vi) 10^4^×10^5^, NS. Results are representative of two independent experiments.

In initial studies we investigated the development of *T. cruzi* parasitemia in wild type C57BL/6 mice challenged i.p. with different doses of trypomastigotes (10^2^, 10^3^, 10^4^ or 10^5^ parasites per mouse). As shown in figure 1, infection with 10^2^ parasites generated a parasitemia that could be first detected at day 8 and peaked at day 11 post challenge. Doses of 10^3^, 10^4^ or 10^5^ parasites hastened the initial detection of parasitemia to days 5, 4 and 3, respectively. The peak parasitemia in mice receiving the highest parasite doses was also earlier, at days 9, 8 or 5 respectively. The magnitude of the peak parasitemias between the different mice groups was not significantly different when comparing groups of mice infected with 10^5 ^or 10^4^. Similarly, no difference was found when comparing groups of mice infected with 10^3^ or 10^2^. Mice infected with 10^3^ parasites presented a peak parasitemia lower (*P*<0.05) than the mouse group infected with 10^4^ but not with 10^5 ^parasites. In repeated experiments, mice infected with 10^2^ parasites presented a peak parasitemia lower than mouse groups infected with 10^4^ or 10^5 ^(*P*<0.01 in both cases).

**Figure 2:**
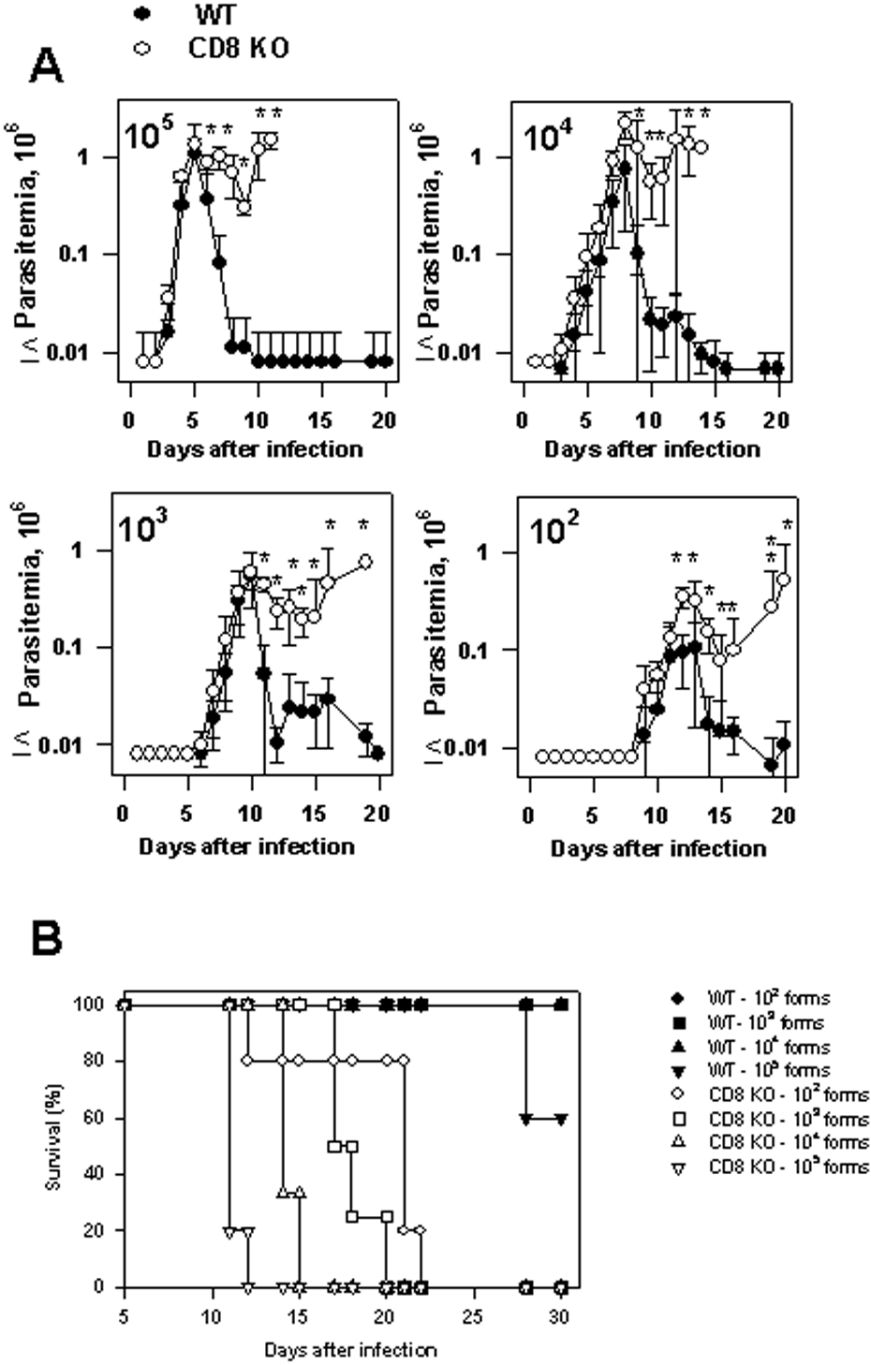
Infection in WT C57BL/6 or CD8 KO mice challenged with different doses of *T. cruzi.* Groups of WT C57BL/6 or CD8 KO were infected i.p. with 10^2^,10^3^, 10^4^ or 10^5^ bloodstream trypomastigotes of the Y strain of *T. cruzi*. (A) Course of infection, estimated by the number of trypomastigotes per mL of blood. Results represent the mean values of 4–5 mice±SD. The parasitemias of WT C57BL/6 or CD8 KO mice were compared by One-way Anova. Asterisks denote statistically significant differences (*P*<0.05). (B) Kaplan-Meier curves for survival of WT C57BL/6 or CD8 KO infected mice with different doses of parasites. Statistical analyses were performed using LogRank test comparing the different mouse groups. Initially, we compared groups of WT C57BL/6 infected with different doses. The results of the comparison showed no statistically significant differences among them. Subsequently, we compared WT C57BL/6 or CD8 KO infected with each different dose of parasites. The results of the comparison showed statistically significant differences in between C56BL/6 or CD8 KO challenged with each parasite dose (*P*<0.0001, in all cases). Finally, statistical analyses were performed comparing the groups of CD8 KO infected with different doses. The results of the comparison were as follows: i) CD8 KO 10^2^×CD8 KO 10^3^ (*P* = 0.0025); ii) CD8 KO 10^2^×CD8 KO 10^4^ (*P* = 0.0046); iii) CD8 KO 10^2^×CD8 KO 10^5^ (*P* = 0.0016); iv) CD8 KO 10^3^×CD8 KO 10^4^ (*P* = 0.01); v) CD8 KO 10^3^×CD8 KO 10^5^ (*P* = 0.0035); vi) CD8 KO 10^4^×CD8 KO 10^5^ (*P* = 0.0082).

In view of these results, we concluded that there is an inverse relationship between parasite inoculum and the timing of the development of *T. cruzi* parasitemia, a feature that had not been described. In addition, we observed that the peak parasitemia did not differ significantly among groups of mice injected with 10^3^, 10^4^, or 10^5 ^parasites. Mice infected with 10^2^ parasites consistently presented lower peak parasitemia than animals challenged with much higher parasite doses (10^4^ or 10^5^ parasites per mouse). In spite of the fact that the parasitemia reached the peak earlier when the parasite inoculum was increased, these mice were capable of controlling the infection and survived the challenge. Because in this mouse model of infection CD8^+^ T cells are critical for survival [13], the fact that animals injected with increasing parasite inoculum survived infection suggested that they were able to develop protective immunity in spite of increasing the infective dose. To determine whether protective immunity dependent on CD8^+^ T cells could indeed be developed in these animals, we compared the parasitemia and mortality of wild type and CD8 KO mice following infection with different doses of parasites. As shown in figure 2A, when comparing infected wild type and CD8 KO mice, the ascendant part of the curve of parasitemia was not significantly different. These results indicated that CD8^+^ T cells were not important for parasite control during that period regardless of the parasite dose used for challenge. After the day of the peak parasitemia, wild type mice rapidly controlled the number of parasites in the blood. In sharp contrast, CD8 KO mice were unable to control the parasitemia, became ill and eventually died.

When we compared the timing of death of each group of CD8 KO mice, we observed an inverse relationship between the size of parasite inoculum and the timing of death. Statistical analysis revealed a significant difference among the groups of infected CD8 KO mice (*P*≤0.01 in all cases, Fig. 2B). As wild type mice survived, these results clearly confirmed the importance of CD8^+^ T cells as a protective mechanism in mice infected with different doses of parasite. These results also suggest that the activation of protective CD8^+^ T cells in wild type C57BL/6 mice takes place earlier as the parasite inoculum is increased.

**Figure 3:**
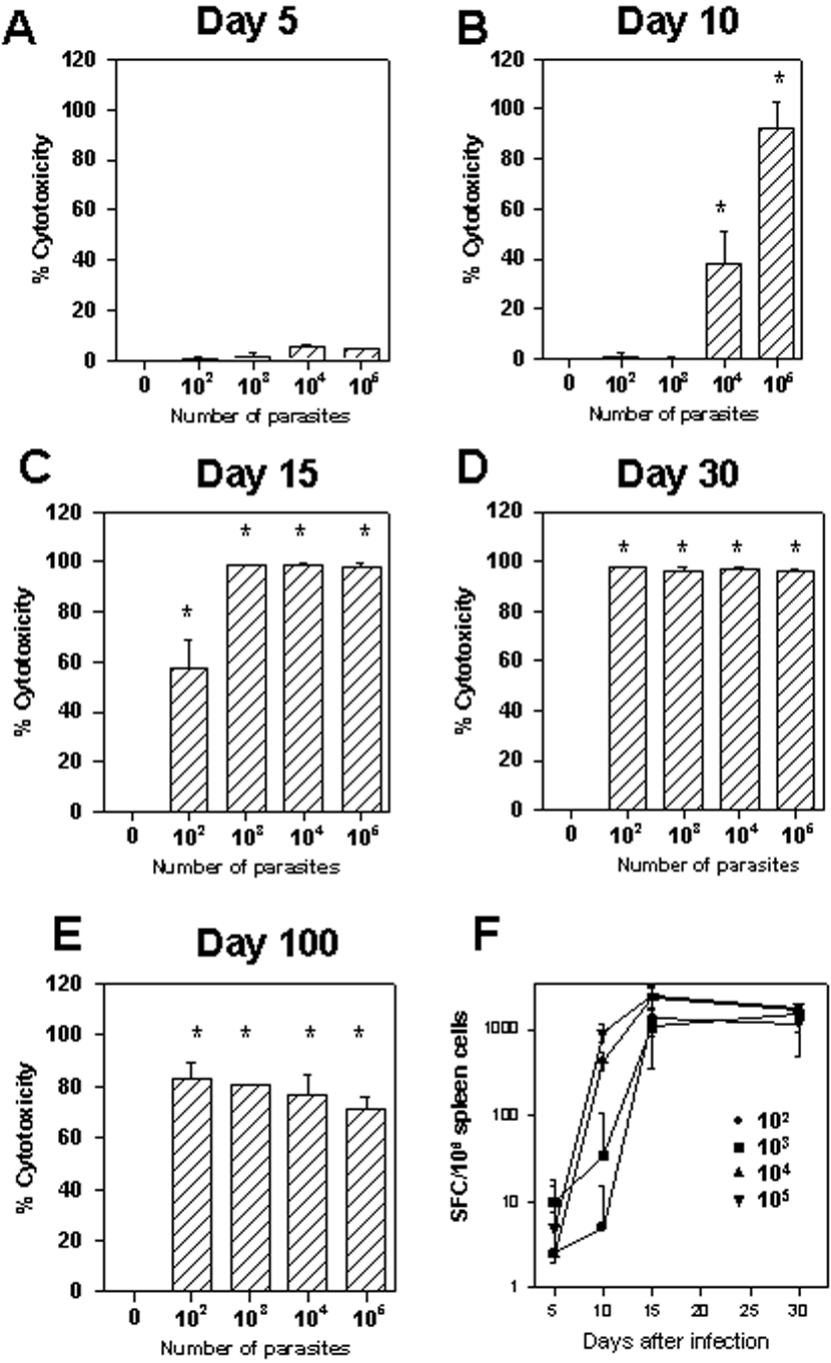
Kinetics of specific CD8^+^ T-cell mediated immune responses following challenge with *T. cruzi.* Groups of C57BL/6 mice were challenged or not i.p. with 10^2^,10^3^, 10^4^ or 10^5^ bloodstream trypomastigotes of the Y strain of *T. cruzi*. Panels A to E - At the indicated days, the *in vivo* cytotoxic activity against target cells coated with peptide VNHRFTLV was determined as described in the Methods Section. The results represent the mean of 4 mice±SD per group. Asterisks denote statistically significant differences when we compared *T. cruzi* challenged with control mice (*P*<0.05). Panel F- At the indicated days, IFN-γ producing spleen cells specific to the peptide VNHRFTLV were estimated by the ELISPOT assay. The results represent the mean number of peptide-specific spot forming cells (SFC) per 10^6^ splenocytes±SD (n = 4). Results are representative of two or more independent experiments.

To determine whether there was an inverse relationship between the parasite inoculum used for infection and the timing of expansion of specific CD8^+^ cytotoxic T cells, we characterized the kinetics of effector CD8^+^ T cell development. For this purpose we used a functional cytotoxic assay which measures the *in vivo* elimination of target cells coated with peptide VNHRFTLV [13]. The phenotype of effector cells mediating peptide-specific *in vivo* cytotoxicity was established earlier as being CD8^+^ T cells [13]. As shown in Fig. 3A, at day 5 post-infection, none of the mouse groups presented peptide-specific cytotoxicity. At day 10 day post-infection, we observed a direct correlation between the size of parasite inoculum and intensity of the *in vivo* cytotoxicity (Fig. 3B). By day 15^th^, groups of mice infected with 10^3^, 10^4^ or 10^5^ parasites had reached their maximum cytotoxicity (close to 100% specific lysis). In contrast animals infected with 10^2^ parasites still displayed only ∼50% cytotoxic activity (Fig. 3C). By day 30^th^ post-infection, the *in vivo* cytotoxicity reached frequencies close to 100% in all mouse groups (Fig. 3D). The *in vivo* cytotoxicity continued at a high level in all infected groups until tested 100 days after challenge (Fig. 3E).

IFN-γ ELISPOT assays confirmed that at day 5 post-infection few peptide-specific T cells were detected in mice infected with increasing doses of *T. cruzi*. A direct correlation between the size of parasite inoculum and the number of peptide-specific cells was clearly evident by day 10 post-infection. By day 15^th^ or 30^th^, in all mice groups we detected a high frequency of IFN-γ producing specific T cells (Fig. 3F).

**Figure 4:**
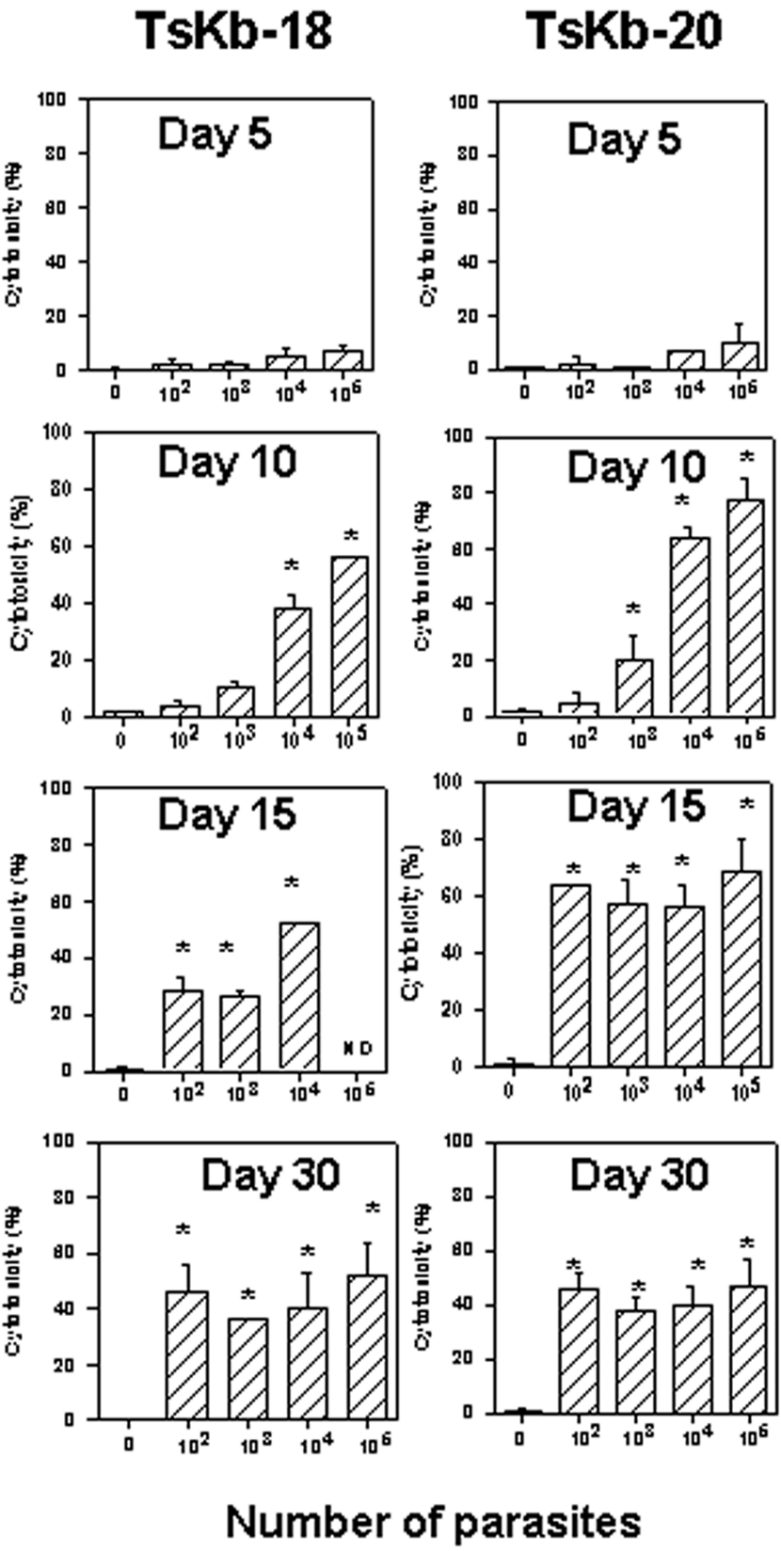
Kinetics of CD8^+^ T-cell mediated immune responses specific for sub-dominant epitopes in C57BL/6 mice. Groups of C57BL/6 mice were challenged or not i.p. with 10^2^,10^3^, 10^4^ or 10^5^ bloodstream trypomastigotes of the Y strain of *T. cruzi*. At the indicated days, the *in vivo* cytotoxic activity against target cells coated with peptide TsKb-18 or TsKb-20 was determined as described in the Methods Section. The results represent the mean of 4 mice±SD per group. Asterisks denote statistically significant differences when we compared *T. cruzi* challenged with control mice (*P*<0.05). ND = Not done. Results are representative of two or more independent experiments.

To evaluate whether the results described above could also be extended to other parasite epitopes, we evaluated the kinetics of the *in vivo* cytotoxicity specific for two other sub-dominant epitopes (TsKb-18 and TsKb-20, ref. 14). We found that the kinetics of cytotoxicity for both sub-dominant epitopes was also dependent on the dose of parasites used for the challenge (Fig. 4).

**Figure 5:**
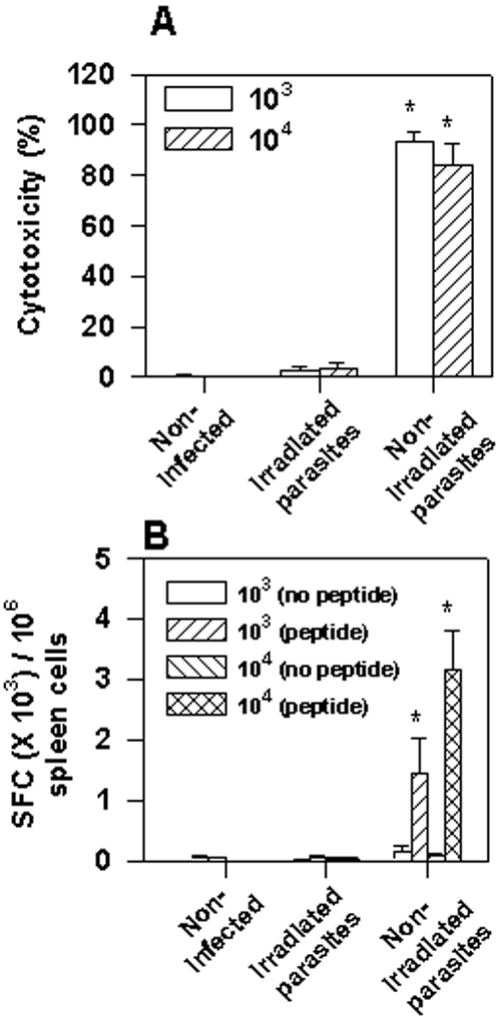
Specific cytotoxicity in C57BL/6 mice challenged with irradiated or non-irradiated trypomastigotes of *T. cruzi.* Groups of C57BL/6 mice were challenged or not i.p. with 10^3^ or 10^4^ irradiated or non-irradiated bloodstream trypomastigotes of the Y strain of *T. cruzi*. A) Fifteen days after challenge, the *in vivo* cytotoxic activity against target cells coated with peptide VNHRFTLV was determined. The results represent the mean of 4 mice±SD per group. B) Fifteen days after challenge, IFN-γ producing spleen cells specific to the peptide VNHRFTLV were estimated by the ELISPOT assay. The results represent the mean number of SFC per 10^6^ splenocytes±SD (n = 4). Asterisks denote statistically significant differences (*P*<0.05) when we compared mice challenged with irradiated or non-irradiated trypomastigotes of *T. cruzi*. Results are representative of two independent experiments.

The results presented above established an inverse relationship between the parasite inoculum and the timing of cytotoxic CD8^+^ T cells differentiation and proliferation. However, it was not clear whether this event was in fact dependent on parasite multiplication or depended solely on dose of parasite used for challenge. To address this question, we challenged mice with irradiated or non-irradiated parasites. Irradiated parasites maintain their viability as assessed by their motility and capacity to infect host cells *in vitro*. However, they are unable to multiply (*in vitro* or *in vivo*) and establish an infection as determined by absence of parasitemia. Mice challenged with 10^3^ or 10^4^ irradiated parasites did not develop detectable *in vivo* cytotoxic activity or IFN-γ producing cells (Fig. 5A and 5B, respectively). In contrast, animals challenged with 10^3^ or 10^4^ non-irradiated parasites developed strong cytotoxic responses and peptide-specific IFN-γ producing cells (Fig. 5A and 5B, respectively). This result suggested that parasite replication was indeed an important factor to promote differentiation and proliferation of cytotoxic T cells.

**Figure 6:**
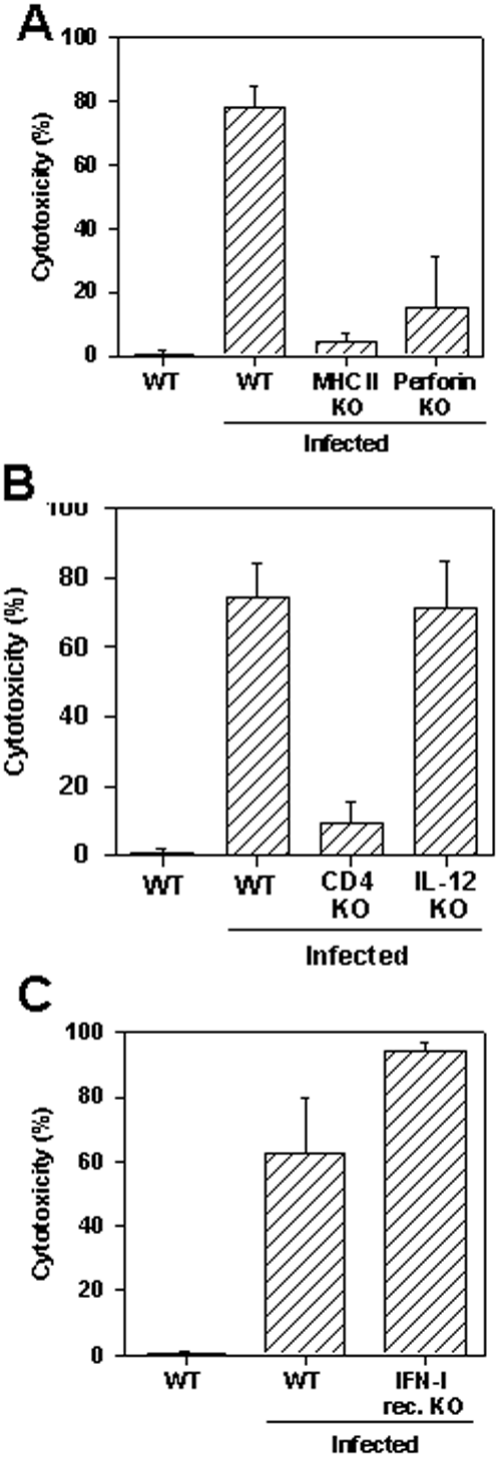
Specific cytotoxicity in WT or genetically deficient mice challenged with *T. cruzi.* Groups of WT C57BL/6 (n = 4), WT 129 mice (n = 4), MHC-II KO (n = 4), perforin KO (n = 8), CD4 KO (n = 4), IL-12 KO (n = 4), and IFN-I receptor KO (n = 4) were challenged or not i.p. with 10^5^ bloodstream trypomastigotes of the Y strain of *T. cruzi*. Ten days after challenge, the *in vivo* cytotoxic activity against target cells coated with peptide VNHRFTLV was determined. The results represent the mean of the above indicated number of mice±SD per group. The *in vivo* cytotoxicity was compared by One-way Anova and Tukey HSD tests. The results of the comparisons were as follows: i) WT C57BL/6×MHC-II KO (*P*<0.01); ii) WT C57BL/6×Perforin KO (*P*<0.01); iii) WT C57BL/6×CD4 KO (*P*<0.01); iv) WT C57BL/6×IL-12 KO (NS); v) WT 129×IFN-I receptor KO (NS). Results are representative of two or more independent experiments.

Because certain genetically deficient mice are highly susceptible to *T. cruzi* infection, and die before specific CD8^+^ T cells could be detected, it is difficult to study the importance that certain cells/molecules may have on proliferation and development of effector functions of CD8^+^ cytotoxic T cells following *T. cruzi* infection. However, the fast development of cytotoxic T cells observed in mice infected with large doses of *T. cruzi* (10^5^ parasites per mouse), allowed us to study some of the molecules of the immune system that could play an important role in the development of protective CD8^+ ^cytotoxic T cells. Using this strategy, we were able to study whether genetically deficient mice lacking MHC-II, CD4, IL-12, perforin or IFN-I receptor were capable of developing specific CD8^+^ cytotoxic T cell responses. We found that MHC-II or CD4 KO mice developed negligible levels of specific cytotoxicity *in vivo* (Fig. 6A and B, respectively). In contrast, IL-12 KO or IFN-I receptor KO mice developed normal levels of specific cytotoxicity *in vivo* (Fig. 6A and 6C, respectively). Cytotoxicity mediated by CD8^+^ T cell responses in Perforin KO mice were significantly reduced (∼75%) compared to control wild type animals. The results of these studies clearly indicate that MHC-II and CD4 are key molecules for the induction of an effective cytotoxic CD8^+^ T cell response following *T. cruzi* infection.

## Discussion

Sufficient amounts of pathogen-derived adjuvant to mature APC and antigen to trigger T cells are possibly among the critical steps required for the differentiation and proliferation of CD8^+^ cytotoxic T cells. However, during infection with different pathogens, it is not clear the point at which the amount of adjuvant and antigen reach the necessary threshold to induce APC maturation and T cell activation. Considering that in most cases the initial pathogen inoculum is limited, pathogen multiplication may be a critical step in this process. In our experimental model, after infection with viable irradiated parasites of *T. cruzi*, we were unable to detect differentiation and proliferation of cytotoxic CD8^+^ T cells (Fig. 5). This result suggests that *T. cruzi* multiplication is critical to generate sufficient amounts of parasite adjuvant for the maturation of APC, and antigens for T cell activation. Our result contrasts with the data published using sporozoites of *P. yoelii*. In this case, relatively small doses (10^4^–10^5 ^parasites per mouse) of non-replicating radiation-attenuated parasites efficiently prime effector/protective CD8^+^ T cells [16]. As for *Listeria*, non-replicating irradiated bacterias were also used during priming of effector/protective CD8^+^ T cells. However, large doses of bacteria (10^9^ per mouse) were employed. This inoculum size of radiation-attenuated bacterias was considerably higher than the inoculum of 10^4^ non-irradiated bacterias per mouse used to prime protective CD8^+^ T cells [17].

While the notion that the timing of accumulation of pathogen-derived antigen/adjuvant may influence the kinetics of expansion of effector CD8^+^ T cells appears to be reasonable, few experimental models provide an opportunity to evaluate this important aspect of the CD8^+^ T cell response. Our initial observation that increasing doses of *T. cruzi* caused an acceleration of *T. cruzi* parasitemia without increasing mouse susceptibility provided a suitable experimental model to study this issue. As observed with *T. cruzi* infection, an inverse relationship was described between the inoculum size of influenza virus and the timing of accumulation of viral load in the lung. However, in this experimental model, the rapid increase in the viral load augmented the apoptosis of CD8^+^ T cells mediated by Fas/FasL interaction, causing a reduction in the *in vivo* cytotoxicity mediated by CD8^+^ T cells and and increased death rate in mice which received larger viral doses [18]. Differently, in our *T. cruzi* model we observed that increasing the size of parasite inoculum accelerated the parasitemia and the timing of differentiation and expansion of cytotoxic CD8^+^ T cells (Fig. 3). However, the different doses of parasite did not modified the final magnitude of the specific CD8^+^ T cell response as measured by the *in vivo* cytotoxicity or the ELISPOT assay (Fig. 3). Therefore, we concluded that differently to the influenza virus system, no inhibitory activity was generated in *T. cruzi* infection by the fast pace of parasite adjuvant/antigen accumulation in C57BL/6 mice.

The curves of parasitemia observed in WT C57BL/6 or CD8 KO mice challenged with different parasite doses, strongly suggest that protective CD8^+^ T cells are important for mouse survival only after the parasitemia reached a peak (Fig. 1 and 2). Until that day, the amounts of parasites in the blood of both, WT C57BL/6 and CD8 KO mice, were similar (Fig. 2, ref. 13). After the peak parasitemia, a rapid reduction in the number of blood parasites was observed in WT mice while CD8 KO mice failed to control parasite growth, became severely ill, and eventually died. Confirmation of the importance of CD8^+^ T cells in the period after the peak parasitemia was obtained in experiments in which we estimated the presence of cytotoxic T cells *in vivo*. For example, while the peak parasitemia of mice challenged with 10^5^ parasites was reached 5 days post-infection, no peptide-specific cytotoxicity was detected at that day (Fig. 3A). However, 5 days later, the *in vivo* cytotoxicity was already 100% indicating that specific CD8^+^ T cells expanded vigorously during that period. Essentially the same sequence of events is observed in mice challenged with different doses of parasites. Based on these observations, we concluded that following challenge of naïve hosts with parasites of the Y strain of *T. cruzi,* the differentiation and expansion of splenic antigen-specific effector CD8^+^ T cells occurs after the peak parasitemia. These results are in close agreement with the data published by us and others using 4 different *T. cruzi* epitopes in two different mouse strains [13], [14]. We consider that our observations are consistent with the interpretation that the amount of *T. cruzi* antigen available before the peak of parasitemia is limited. When the parasitemia reaches its peak, the threshold for the level of adjuvant/antigen requirement may be achieved and only then, the triggering and fast activation of naïve CD8^+^ T cells may occur. Studies performed in mice infected with LCMV or *L. monocytogenes* described comparable timing for expansion of specific CD8^+^ T cells i.e., the peak of viral or bacterial numbers occurs approximately 2–3 days after infection. The peak of CD8^+^ T cell response was approximately 5 days later at day 7–8^th^ post-infection [1], [2].

In the last part of our study, considering that a rapid induction of *T. cruzi*-specific CD8^+^ T cells occurred after administration of a large inoculum of parasites, we evaluated the importance that certain molecules/cells may have on differentiation/proliferation and effector function of specific CD8^+^ T cells following *T. cruzi* infection. For this purpose, we used genetically deficient mouse strains that are described as highly susceptible to infection with *T. cruzi* such as MHC-II KO, CD4 KO, IL-12 KO and perforin KO [13], [19]–[22]. MHC II KO or CD4 KO mice failed to develop peptide-specific cytotoxicity. We therefore concluded that MHC II-restricted CD4^+^ T cells are important for the maturation and/or expansion of *T. cruzi* specific cytotoxic CD8^+^ T cells.

Our results indicating that CD8^+^ T cells responses against *T.cruzi* are critically dependent on CD4^+^ T cells differ from most pathogens. Following viral or bacterial infections, the maturation and expansion of specific CD8^+^ T cells are not critically dependent on CD4^+^ T cells [23]–[29]. Similarly, CD4^+^ T cells are not required for the initial expansion of CD8^+^ T cells specific for epitopes expressed by the protozoan parasites *P. yoelii* or *T. gondii* [11], [30], [31]. The precise role for MHC II-restricted CD4^+^ T cells during the process of CD8^+^ T cell activation in our model has yet to be investigated. An intriguing possibility is that CD4^+^ T cells can license dendritic cells for the activation of highly cytotoxic CD8^+^ T cells detected by an *in vivo* assay [32].

Using IL-12 or IFN-I receptor KO mice, we observed that neither IL-12 nor IFN type I are critically important for the efficient maturation and expansion of *T. cruzi*-specific cytotoxic CD8^+^ T cells. Our results contrasts with previous observation that IL-12 can provide an important third signal that, in addition to the engagements of TCR-MHC and CD28-B7, it could provide an optimal environment for the efficient cytotoxic CD8^+^ T cells differentiation and expansion [33]–[35].

Perforin KO mice were also severely impaired in their ability to eliminate peptide-coated targets *in vivo*. The low level killing detected in the absence of perforin may represent the contribution of perforin-independent killing mechanisms. Considering our previous results indicating that perforin KO mice are highly susceptible to infection with parasites of the Y strain of *T. cruzi*, we propose that the perforin-dependent granule exocytosis pathway represent an important mechanism of protection against *T. cruzi* infection. These results are in agreement with some viral models describing perforin as a key molecule for resistance against viral infection and mediating *in vivo* lysis of peptide-coated target cells [36], [37]. However, they differ with the observations made for other protozoan parasites in which perforin KO mice have been shown to develop protective CD8^+^ T cell mediated immunity [38]–[40].

In summary, our study provides new insights regarding the requirements for the differentiation and expansion of cytotoxic CD8^+^ T cells during experimental infection with a human protozoan parasite. Using this experimental model, we determined the importance of parasite load and MHC-II restricted CD4^+^ T cells for the maturation and expansion of highly cytotoxic CD8^+^ T cells. Also, it established an important role for perforin as a mediator of the *in vivo* cytotoxicity against parasite-infected cells.
